# Midwives' experiences of traumatic births: A systematic review and meta-synthesis

**DOI:** 10.18332/ejm/138197

**Published:** 2021-07-26

**Authors:** Ruveyde Aydın, Songül Aktaş

**Affiliations:** 1 Department of Birth, Women Health and Disease Nursing, Faculty of Health Sciences, Karadeniz Technical University, Trabzon, Turkey

**Keywords:** midwife, traumatic birth, posttraumatic stress, secondary trauma, qualitative research, meta-synthesis

## Abstract

**INTRODUCTION:**

Midwives experiencing traumatic births are emotionally affected by this process, lose their self-confidence, and may intend to leave the profession. This study aims to carry out a meta-synthesis of current qualitative research exploring the experiences of midwives witnessing traumatic births.

**METHODS:**

The meta-synthesis consisted of 18 full-text studies in English, obtained from PubMed, Scopus, Web of Sciences, Cumulative Index of Nursing and Allied Health Literature (CINAHL), EMBASE, and PsycINFO databases. The results of the studies were analyzed using the thematic analysis technique. The study includes qualitative, mixedmethod, and full-text studies published between 2000 and 2020 that explored the experiences of midwives and obstetric nurses witnessing birth trauma.

**RESULTS:**

The thematic analysis identified seven themes: post-traumatic feelings, posttraumatic stress symptoms, the impact of trauma on professional values, social support, learning from experience, legal process, and reflection of emotions of women experiencing traumatic birth on the midwife.

**CONCLUSIONS:**

Midwives who witnessed traumatic birth were mostly emotionally affected. They lost their self-confidence and intended to leave their profession. They emphasized the importance of peer support through which they could share their experiences after trauma. Psychological education should be provided to midwives who witness the trauma by specialists, and midwives should be strengthened against the effects of trauma in terms of both the institutional policies where the birth takes place and midwifery-specific legal policies.

## INTRODUCTION

A traumatic event is defined as an unpleasant event that may result from an injury, violence, or emotional shock^1^. It does not have to occur due to a major disaster, but it carries an elevated risk of the stress response^2^. It suddenly occurs without any symptoms and weakens the individual’s defence mechanisms. Thus, it causes individuals to experience loss of control and fear^3^. Although childbirth is considered to be a positive life experience, adverse events occurring during the intra-natal period may increase the risk for maternal, fetal, and neonatal mortality and morbidity^4^. Therefore, complications occurring during childbirth can create a situation that fits the post-traumatic stress criteria of the American Psychiatric Association Diagnostic and Statistical Manual^5^. While it is mothers and/or fetus/neonate who experience the trauma at birth, midwives who care for mothers and communicate with them emotionally and empathically are also witnessing this trauma. The two most significant components of professional midwifery care at birth are compassion and empathy. There is a strong bond between midwives, birthing women, and the birth process, in which intense emotions are experienced^1^. Such strong feelings increase the risk and severity of the emotional stress felt by the midwife in case of an undesirable or traumatic circumstance. This may even result in the midwife experiencing secondary traumatic stress disorder^6^.

According to DSM-V, Post-Traumatic Stress Disorder (PTSD) is triggered by an important traumatic event and develops as a state of excessive arousal in a person, an avoidance of stimuli that reminds the person of the trauma, and re-experiencing the traumatic event through dreams, nightmares, and flashbacks. PTSD is a mental issue in which a person experiences symptoms for at least one month following a traumatic event^5^. Secondary traumatic stress disorder (STSD), on the other hand, occurs as a result of witnessing the trauma and develops symptoms similar to those of PTSD such as sad dreams, constant anxiety, anger, depression, hopelessness, irritability, discomfort stemming from remembering the event, difficulty in concentrating and insomnia^7^. Studies examining the experiences of midwives following the traumatic event have focused on STSD and PTSD. A study conducted in the Netherlands reported that 13% of midwives feel traumatized following birth trauma, and 2.2% of midwives experience STSD^8^. Another study in the United States, Beck and Gable^1^ found that 35% of midwives had PTSD. Wallbank et al.^9^ also indicated that midwives often felt symptoms of PTSD after a neonatal death, miscarriage, and difficult birth, and consequently avoided similar events as they constantly thought about the trauma. Although exposure to traumatic events may not always fit PTSD criteria, it could cause symptoms that adversely affect the psychological health of the individual^10^.

Traumas experienced by midwives during the birth process may lead to burnout, unmet care, emotional fatigue, deterioration of interpersonal relations, increase in conflict, dejection, and other similar problems^11^. Rice and Warland^6^ describe the experiences of midwives witnessing traumatic births with ‘guilt, weakness, and responsibility’. Another study noted that midwives had difficulty in providing professional care and maintaining professional values for the mother experiencing trauma and suffered from witnessing the traumatic birth^1^. Calvert and Benn^3^ also reported that midwives experienced such intense feelings as loss, grief, loss of self-confidence, and violence after a traumatic birth that they needed support. Evidence shows that midwives experience guilt, failure, frustration, shame, intend to leave the profession, and PTSD^10,12,13^. Hence, it seems critical to explore how midwives’ experiences of traumatic births could adversely affect their mental health and the quality of midwifery care during childbirth and the early postpartum period employing a meta-synthesis of qualitative studies focusing on the experiences of midwives witnessing traumatic births. The study aimed to conduct a meta-synthesis of current qualitative research exploring the experiences of midwives witnessing traumatic births. The research question was: ‘What are the experiences of midwives witnessing a traumatic birth?’.

## METHODS

### Study design

A meta-synthesis was carried out to synthesize and interpret qualitative research about the experiences of midwives witnessing traumatic births.

### Search strategy

This review was limited to articles published in English between 2000 and 2020. First, we scanned the relevant literature in PubMed, Scopus, Web of Sciences, CINAHL, EMBASE, and PsycINFO databases using the following keywords:

*Population*: Midwife OR midwives OR nurse-midwives OR nurse-midwife OR nurse-midwives OR midwifery OR obstetric nurse OR nurse in the delivery room.

*Outcomes:* Trauma OR secondary trauma OR posttraumatic stress OR post-traumatic stress OR secondary traumatic stress OR Second victim.

*Study design:* Not quantitative, not review. Finally, we performed a general search on Google Scholar with keywords and obtained related studies.

### Inclusion and exclusion criteria

The study covers the qualitative, mixed-method, and fulltext studies published in English between 2000 and 2020 that explored the experiences of midwives and obstetric nurses on witnessing birth trauma. Case studies, book chapters, abstracts, reviews, theses, editorials, and studies investigating the traumatic experience of other health professionals such as obstetricians and women who give birth were excluded from the review.

### Rating process

**Figure 1:**
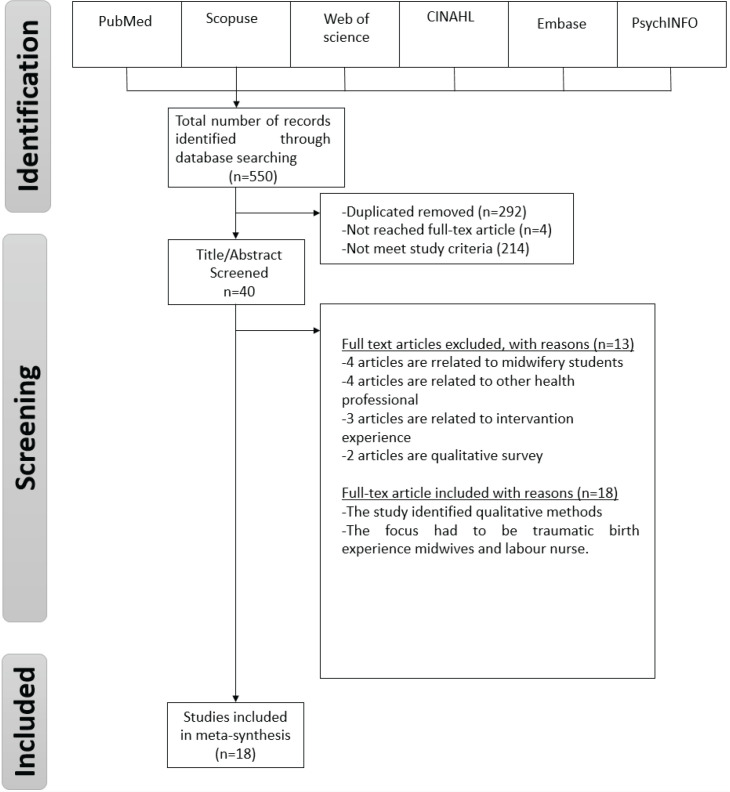
The flow chart showing the results of the search strategy and a description of the studies under review

As a result of the literature scanning, 550 studies were obtained, of which 292 were duplicates and 4 could not be reached in full text. The remaining 214 studies did not meet the research criteria according to their titles and abstracts. The detailed review of other 40 studies showed that 4 studies were related to midwifery students, 4 were related to other health professionals, 3 articles were related to intervention experiences, 2 studies were a qualitative survey study, 1 was conducted over the phone, and 1 was related to post-education experiences. After excluding all the irrelevant articles, 18 studies were left meeting the research criteria (Figure 1).

### Data extraction and quality assessment

Two researchers (RA, SA) scanned the articles by reading their titles and abstracts. An opinion exchange was made by the researchers when a disagreement arose regarding the inclusion criteria. The number of studies included in the meta-synthesis is shown in Figure 1. The quality evaluation of the articles was conducted independently by the researchers. In case of doubt or incomprehensibility, they reevaluated the articles.

The researchers assessed each of the qualitative studies to determine their quality using the checklist developed by Kmet et al.^14^. The checklist consists of 10 questions evaluating the purpose, the research question, data collection and analysis method, supporting the results with the findings, the context, validity, and reliability of the research. The scoring of the checklist is evaluated as Yes (2), Partial (1), and No (0), with a range in points in the checklist 0–20. The total score for each article is calculated by summing the total score from 10 items and dividing it by 20. In this study, the total score of 10 items was divided by 20 and the quality of the articles was given as a percentage. The quality of the articles varied between 80% and 100% (Supplementary file).

### Data synthesis

The thematic analysis approach including six steps by Braun and Clarke^15^ was used for the analysis of the data. We first read the data to become familiar with them and identify remarkable statements about the participants’ experiences. In the second step, initial codes were generated by putting all the common expressions together, and then listed and organized into meaningful groups. In the third step, themes were generated by bringing together similar initial codes. The generated themes were subsequently reexamined, defined and named in the fourth step. In the fifth step, the themes were identified, and which aspects of the data are effective in identifying the themes were clarified, followed by reporting the analysis. The results of the analysis were checked by an external person.

### Rigor and trustworthiness

As in all scientific studies, qualitative research also deals with producing valid and reliable information within the ethical rules. To ensure the validity and reliability of the meta-synthesis studies, the research problem, data scanning databases, keywords, the inclusion criteria for the meta-synthesis, the synthesis of the data, the duration of synthesis, and by whom it was made, must be clearly stated^16^. All the steps mentioned above were carefully followed in this review.

## RESULTS

According to systematic search analysis, 18 studies meeting the research criteria were included in the review. The studies were mostly published between 2013 and 2020, six had the mixed-method, and 12 had the qualitative research design. A semi-structured interview form was applied in all the studies. Generally, thematic and content analyses were used in the studies. The number of samples determined using the purposive sampling method varied between six and twenty-two. The studies have been published in journals of midwifery, birth, nursing, women’s health, and healthcare. Details of the articles are presented in Table 1.

**Table 1:** Characteristics of studies involved in meta-synthesis

*No.*	*Authors*	*Date*	*Sample size*	*Journal*	*Methods*	*Data collection*	*Data analysis*
1	McCool et al.^24^	2009	22	Health Care Women Int	Qualitative	Semi-structured interview form	Content analysis
2	Goldbort et al.^7^	2011	9	Mcn-Am J Matern-Chil	Phenomenology	Semi-structured interview form	Thematic analysis
3	Halperin et al.^18^	2011	18	J Midwifery Women’s Health	Qualitative	Semi-structured interview form	Content analysis
4	Beck and Gable^1^	2012	464	Obstet Gynecol Neonatal Nurs	Mixed method	Semi-structured interview form	Content analysis
5	Lindberg et al.^2^	2013	8	Br J Midwifery	Qualitative	Semi-structured interview form	Content analysis
6	Rice and Warland^6^	2013	10	Midwifery	Descriptive qualitative	Semi-structured interview form	Thematic analysis
7	Edqvist et al.^12^	2014	13	BMC Pregnancy Childbirth	Phenomenology	Semi-structured interview form	Thematic analysis
8	Beck et al.^4^	2015	473	J Midwifery Womens Health	Mixed method	Semi-structured interview form	Content analysis
9	Calvert and Benn^3^	2015	16	Int J Childbirth	Narrative science	Semi-structured interview form	Eclectic approach
10	Sheen et al.^11^	2016	421	Midwifery	Mixed method	Semi-structured interview form	Thematic analysis
11	Schrøder et al.^13^	2016	1237	Acta Obstet Gynecol Scand	Mixed method	Semi-structured interview form	Thematic analysis
12	Fontein-Kuipers et al.^10^	2018	106	Eur J Midwifery	Mixed method	Semi-structured interview form	Content analysis
13	Toohill et al.^20^	2019	199	Women Birth	Mixed method	Semi-structured interview form	Content analysis
14	Patterson et al.^22^	2019	6	Midwifery	Phenomenology	Semi-structured interview form	Thematic analysis
15	Christoffersen et al.^19^	2020	33	Midwifery	Qualitative	Semi-structured interview form	Content analysis
16	Nyberg et al.^25^	2010	15	Sex Reprod Health	Qualitative	Semi-structured interview form	Content analysis
17	Huang et al.^26^	2019	14	Asian Nurs Res	Qualitative	Semi-structured interview form	Thematic analysis
18	Cankaya et al.^23^	2020	29	J Eval Clin	Qualitative	Semi-structured interview form	Content analysis

Seven themes were identified in the thematic analysis: post-traumatic feelings, post-traumatic stress symptoms, the impact of trauma on professional values, social support, learning from experience, legal process, and reflection of emotions of women experiencing traumatic birth on the midwife (Table 2).

**Table 2:** Themes and sub-themes

*Themes*	*Sub-themes*
**Post-traumatic feelings**	Shocked
	Crying
	Feeling the chaos
	Feeling helpless
	Being disrespected as a midwife
	Powerless
	Feeling sadness
	Feeling guilty
	Feeling shame
	Feeling like a failure
	Feeling disappointed
**Post-traumatic stress symptoms**	Flashbacks
	Nightmares
	Inability to forget
	Avoidance
**The impact of trauma on professional values**	Loss of confidence in professional practice
	Desire to quit the profession
	Maintenance of professional values
**Social support**	Need for social support
	The importance of social support
**Learning from experiences**	-
**Legal process**	Sadness due to the lawsuit
	Collecting evidence for lawsuit
**Reflection of emotions of women experiencing traumatic birth on the midwife**	-

### Theme 1: Post-traumatic feelings

Post-traumatic feelings of midwives after trauma were identified as ten sub-themes: being shocked, crying, feeling the chaos, feeling helpless, being disrespected as a midwife, feeling powerless, sad, guilty, shame, disappointed, and feeling like a failure (Table 3).

**Table 3:** Sub-themes of Theme 1 ‘Post-traumatic feelings’

*Sub-themes*	*Expression of midwives*
**Being shocked**	*‘The difficulty is both physical and emotional, your blood boils … and there is no blood that goes to the brain, your whole body is paralyzed from shock. I was totally in shock.’ ^17^*
	*‘… I mean she was more shocked than me because she entered the labor room and I said oh my god [name omitted] baby's dead.’ ^11^*
**Crying**	*‘… The doctor and I began to work through tears in our eyes. We just started to quietly cry ...’ ^1^*
	*‘I felt terrible, such a loss. I cried with the woman—that's the way it was—a very deep sadness. The second midwife also cried with us.’ ^17^*
**Feeling the chaos**	*‘What I remember happening is, walking in and everybody's flurried around doing all kinds of stuff. And I noticed that the patient's blood pressure on the monitor machine is very low ... And I just went to the obstetrician... [then] I went to the anesthesiologist, “What can I do for you? What do you need?” ’ ^18^*
**Feeling helpless**	*‘I have thought many times that we do not have good follow-up routines after critical incidents. You feel abandoned, and you have no one to lean on.’ ^19^*
**Being disrespected as a midwife**	*‘I was abused in front of the woman when I had turned off the Syntocinon infusion because of decelerations during contractions.’ ^20^*
**Feeling powerless**	*‘I have stood by helpless and watch [sic] babies die due to my inability to perform a cesarean and my [back up] is 30 minutes out.’ ^4^*
**Feeling sadness**	*‘I was absolutely devastated. Absolutely, I broke down …’ ^11^*
**Feeling guilty**	*‘So that instantly knocks you back into going “hang on a minute”, what's wrong with me, did I make that really bad for the woman? It was just so terrible, I felt so guilty.’ ^6^*
**Feeling shame**	*‘Unfortunately, I think that many midwives feel such shame that they may not want to admit that a sphincter tear has occurred and then do everything to hide it.’ ^2^*
**Feeling like a failure**	*‘There's also I guess for me, there’s almost a sense of failure, and that I've failed this person.’ ^6^*
**Feeling disappointed**	*‘I felt that I had disappointed the family, although it was beyond my control, you know …’ ^11^*

### Theme 2: Post-traumatic stress symptoms

After a traumatic birth, midwives had post-traumatic symptoms such as flashbacks, nightmares, inability to forget, and avoidance. Sub-themes and expressions of midwives are given in Table 4.

**Table 4:** Sub-themes of Theme 2 ‘Post-traumatic stress symptoms’

*Sub-themes*	*Expression of midwives*
**Flashbacks**	*‘Whenever I hear a patient screaming, I will flashback to a patient who had an unmedicated (not even local) cesarean section and to the wailing of a mother when we were coding her baby in the delivery room.’ ^1^*
**Nightmares**	*‘I had nightmares for several weeks after that, wondering about … how that could happen and what it was … It was very difficult from the first few weeks afterward to come to work.’ ^7^* *‘When I go home and close my eyes, I see and feel that terrible incident in my dream. I try to forget, of course … but I even can't sleep …’ ^23^*
**Inability to forget**	*‘I can't forget it. I can still see the lady's face. I can't forget that. I'm not going to forget it.’ ^11^* *‘Time heals but it never goes away completely … I will never forget it … there is a scar … up to today.’ ^10^*
**Avoidance**	*I had a patient die in the delivery room; she was preeclamptic. I felt terrible, depressed, and impotent. I couldn't go into that delivery room for days …’ ^24^*

### Theme 3: The impact of trauma on professional values

The impact of trauma on midwifery professional values was investigated under three sub-themes including loss of confidence in professional practice, intention to leave the profession, and maintenance of professional values. The subthemes and expressions of midwives are given in Table 5.

**Table 5:** Sub-themes of the Theme 3 ‘Impact of trauma on professional values’

*Sub-themes*	*Expression of midwives*
**Losing confidence in professional practice**	*‘Emotionally, this affected me, I lost my confidence, and I suddenly did not want to give my opinion on things. It made me doubt myself.’ ^3^* *‘… I lost my confidence in myself for a while and carried my nervousness about safety to the next birth.’ ^24^*
**Desire to quit the profession**	*‘I thought my standards and the woman's care had been compromised. I just felt I couldn't do that anymore and that is the reason I gave up midwifery.’ ^3^*
**Maintenance of professional values**	*‘I really dreaded going to work after the incident. I wondered how I would react. And of course, the tears came when I talked to those who had the evening shift and those who came on the night shift that night.’ ^19^* *‘When I have them, it feels just like you, I have a crystal ball in my hand, and if I pinch too hard, I can break it, we must be careful I think, not being forceful.’ ^25^*

### Theme 4: Social support

Midwives emphasized that they need post-traumatic support, which is very important. Two sub-themes were identified.

#### Sub-theme 1: Need for social support

Post-traumatic midwives expressed their need for social support as follows:

*‘We need a safe forum to share with our colleagues. If there is an adverse outcome, we are told to keep silent. There is no place to talk to unburden our souls.’ ^1^*

*‘Maternity ward manager is absent. Support after a critical incident depends on which colleagues you work with that day.’ ^19^*

A midwife said that after being traumatized, people were disrespectful towards her and her inability to get support from her colleagues made her feel like a neglected animal.

*‘I talked to the doctor, but I needed my colleagues to support me. People treated me with disrespect. I came to work the next day, and one of the midwives asked me when I would stop causing tears for all the women that I am taking care of because she heard that I was constantly doing this ...’ ^17^*

#### Sub-theme 2: The importance of social support

Some midwives felt lucky for receiving support from their colleagues and expressed their feelings as follows:

*‘I feel very fortunate to be working in an environment where I am always supported as a novice nurse, and I mean I'm working with these people … I think that it is a tremendously gifted position to be in, to be respected as a novice nurse.’ ^18^*

One midwife explained her relief after talking to someone about her post-traumatic experiences as follows:

*‘Once you've talked to somebody about it properly, it feels as if a weight is just lifted off your shoulders, and you can speak about it, and you feel like you, you know you've just got it off your chest and you can sort of move on in a way.’ ^21^*

### Theme 5: Learning from experiences

Midwives explained that past traumatic experiences were a learning process for them:

*‘I think back to that first situation that I had, and I think of how better I responded in that emergency. How much I had grown and how … much more comfortable I felt just… I know that those experiences are important to help us learn ...’ ^7^*

*‘I've used it as a learning tool, I've kind of tried to turn it the other way round and think what I can use from this, and I've used it to regain my confidence, I've used it to cope with similar scenarios, how I deal with those kinds of stressful scenarios …’ ^11^*

### Theme 6. Legal process

The legal process was examined under two sub-themes: sadness due to the lawsuit and collecting evidence for it.

#### Sub-theme 1. Sadness due to the lawsuit

After the traumatic birth, the midwives expressed their sadness due to the lawsuit filed against them with the following:

*‘I was involved in the resuscitation of a baby who did poorly at birth. I had not delivered the baby but saw the family at the birth center for office hours and offered my assistance when resuscitation was needed. I visited the mother and baby in the neonatal intensive care unit, and she thanked me for saving her baby's life. I was shocked when several months later I was named in a lawsuit … I could not eat and sleep. The case was eventually settled. It was all about money.’ ^24^*

*‘The obstetrician and the hospital attempted to apportion blame me during the three years and five separate investigative processes. I was very upset.’ ^20^*

*‘Battling all the time against the system at traumatic childbirth.’ ^22^*

#### Sub-theme 2: Collecting evidence for the lawsuit

It is stated that gathering evidence is very important for the midwifery profession with the possibility of a lawsuit that will be filed after the traumatic birth:

*‘I had a patient who experienced a placental abruption in labor several years ago. Although I knew I had not done anything clinically wrong, I feared that there would be a lawsuit, so I took copious notes to help me remember the clinical facts. Sure enough, the case came to trial several years later; the jury found in my favor. A student studying midwifery should accept today, that one day they could be sued for a poor outcome in practice.’ ^24^*

### Theme 7: Reflection of emotions of women experiencing traumatic birth on the midwife

Some of the midwives stated that women suffered a lot and felt hopeless in traumatic births:

*‘Some birthing women had longer first stage of labor, and they felt discouraged and hopeless. The long labor time and the pressure from other birthing women may make things worse.’ ^26^*

Some of the midwives expressed that attending the funeral is important for them and the family when the baby is lost as a result of traumatic birth:

*‘It was just the parents, the priest, and me. I spent the evening after the funeral with the parents. I think it was as much my needs as for theirs … A good dialogue with the couple in retrospect helps to process the event. It is difficult when you can't communicate with the parent.’ ^19^*

## DISCUSSION

The study aimed to systematically examine the studies exploring the experiences of midwives who witnessed traumatic births. Although all studies have different objectives, they all contributed to the experiences of midwives in traumatic births, and their themes mainly focus on post-traumatic stress disorder symptoms. As a result of the secondary qualitative analysis of the primary qualitative studies, seven themes emerged. These themes were: post-traumatic feelings, post-traumatic stress symptoms, the impact of trauma on professional values, social support, learning from experience, legal process, and reflection of emotions of women experiencing traumatic birth on the midwife.

### Post-traumatic feelings

Midwives in this study experienced negative feelings during and after the trauma. As stated in the sub-themes, midwives felt shocked, guilt, chaos, and helpless, and they cried during the trauma and felt helpless, powerless, sad, guilt, shame, and failure after the trauma^1,2,4,6,10,17,18,23^. A study conducted with 18 midwives in Israel reported that midwives experienced sadness, crying, disappointment, and shock due to the loss of the baby^17^.

In a study in Australia, more than half of midwives felt fear and guilt during or immediately after trauma^27^. In a mixed-method study by Toohill et al.^20^ it was reported that approximately 94% of midwives experienced a traumatic birth, and one in ten midwives experienced fear at birth. The findings of this study are consistent with the literature. The traumatic birth experience of the midwife may increase her/his fear of birth. The midwife’s fear of birth is a risk for decreased quality of midwifery care in the philosophy of maintaining the normality of birth^12^. Female midwives can also be psychologically more sensitive in stressful and traumatic births. The fact that the midwife has experienced the birth itself and the experience of trauma in the birth of her baby are the factors that increase the risk of being negatively affected by the birth trauma^10^.

### Post-traumatic stress symptoms

Birth is defined as a condition with a high potential to fit post-traumatic stress criteria in DSM-V^5^. This study revealed that midwives experienced some of the symptoms of post-traumatic stress (flashbacks, nightmares, inability to forget, avoidance). Negative emotions such as shock, crying, guilt, and helplessness experienced by midwives during and after birth trauma may have been predisposing factors for post-traumatic stress disorder^4,23,24^. As a result of the analysis, it was seen that midwives having PTS symptoms experience birth traumas due to maternal and infant death, shoulder dystocia, and deep perineal tears. Like this study, in the study of Leinweber and Rowe^27^, midwives who cared for women with birth trauma reported that they experienced secondary traumatic stress.

### The impact of trauma on professional values

Negative feelings and post-traumatic stress symptoms experienced by the midwife during and after trauma adversely affect the current and future mental health of the midwife, the quality of midwifery care, and their private and professional lives^17,23,24^. Besides, the literature emphasizes that the midwives may experience post-traumatic stress disorder in cases in which the empathic approach cannot be controlled in midwife–woman relationships, and occupational burnout that may damage the nature of midwifery care^27,28,29^. Trauma witnessed by midwives in this study caused loss of confidence in professional practices, intention to leave the profession, and difficulty in sustaining professional values^1,3,13,17^. Halperin et al.^17^ reported that some midwives quit their jobs after being involved in a birth with a negative outcome and rested for the rest of their lives.

### Social support

If the midwives exposed to trauma are not treated, the mental health of the women that these midwives care for is at risk^4,11,23^. In a qualitative study, non-empathic behaviors, and some applications (such as fundal compression, episiotomy) of midwives^30^ and the negative attitude and ability of midwifery in the previous birth of multigravidas in another study^31^ were found to have an impact on negative birth experience and traumatic birth perception. However, the midwife’s empathic and emotional relationship with women can reduce the risk of trauma. Rice and Warland^6^ emphasized that midwives’ interest in the feelings and emotions of women who give birth may affect the successful management of traumatic births. In this study, some midwives needed social support from their colleagues in the post-traumatic period, which was valuable for them in coping with trauma. Literature also cites that the midwife needs more social support, especially in challenging deliveries requiring medical intervention^21,23^. In traumatic births, the midwife, the birthing woman, the other healthcare workers, and the hospital management should have an empathic relationship. The midwife, who cannot get enough social support from her colleagues, institution, and manager, feels lonely and helpless^23^. This situation leads to the midwife’s inability to use his/her professional capacity sufficiently, professional burnout, a decrease in job satisfaction^7^, and the desire to quit the profession^18^. It should be remembered that adequate social support to the midwives will contribute to their self-confidence in their knowledge and skills to promote normal birth^21^.

### Learning from experiences

Traumatic experiences do not always result in negative consequences. They may contribute to post-traumatic growth such as becoming more confident, and growing personally, spiritually, or professionally^7,32,33^. Following traumatic birth, some midwives experience growth in personal strength, appreciation of life, relating to others, existential and spiritual change, and new possibilities^32,33^. Post-traumatic peer support and personal characteristics affect traumatic growth. Growth in midwives after a traumatic birth experience provides the empathic approach of the midwife to women who experience a traumatic birth, increases the quality of midwifery care, ensures that their experiences are shared with their colleagues, and strengthens professional cooperation^10^. After the trauma, some midwives in this study considered their trauma as something contributing to their learning and awareness^10,18,21,30^. Elmir et al.^32^ stated that midwives in difficult births experienced weaknesses, failures, and pain, but later these experiences turn into positive contributions, which helped them to develop strategies and problemsolving skills to cope with difficult situations and to guide their colleagues. Moreover, post-traumatic growth can reduce fear and increase trust^33,34^. Research has demonstrated that midwives can professionally be stronger, establish a more positive midwife–woman relationship after trauma, and this experience will increase the power to cope with future traumatic events^10,32-34^. One of the midwives in the study by Fontein-Kuipers et al.^10^ expressed the posttraumatic growth after a traumatic birth as follows:

*‘At the end of the day, it allowed me to grow … I wouldn't be where I am right now if it [event] wouldn't have happened … maybe it even made me a better person … a better midwife.’*

As seen in the expression of the midwife, posttraumatic growth can provide learning from experiences and psychological growth.

### Legal process

The legal process after birth trauma can further deteriorate the birth trauma of the midwife^24,31^. This study revealed that the midwife’s sadness due to the lawsuit filed against her or her collecting evidence against the possibility of a lawsuit was a further trauma for her. In countries where midwifery education, relevant associations, and laws, are not strong enough, midwives are at greater risk of birth trauma^20,35^. Besides, the state does not pay compensation insurance for midwives in some countries, so midwives are supposed to pay it^35,36^.

### Reflection of emotions of women experiencing traumatic birth on the midwife

Factors such as prolonged labor, severe perception of birth pain, interventions applied at birth (fundal pressure, episiotomy, etc.), and negative communication of midwives during birth could lead women to perceive birth as traumatic^21,22,25,31^. Haung et al.^26^ determined in their study that prolonged labor, high severity of birth pain, and baby loss as the reasons for the traumatic perceptions of women from the midwives’ perspective.

Midwives experience secondary trauma after birth due to being exposed to the negative attitudes and behaviors of the mother, spouse, and family, reacting to the gender of the newborn baby^25^. In some traditionally structured communities, having a male newborn baby is seen as the continuation and status gain of the lineage. In addition, the misconception that ‘the female chromosomes determine’ the gender of the baby is dominant^28^. When a woman gives birth to a baby girl, her maternity status is ignored, and she is subjected to psychological violence by the family and the environment^21^. In a study conducted in China, midwives explained that women who gave birth to a baby girl are not accepted by their families, and they feel collapsed and experience traumatic birth^22^.

To reframe and make sense of the situation after a traumatic situation, healthcare professionals use emotional (crying, keeping a diary, etc.) and task-oriented (attending a funeral, etc.) coping methods^10,35^. The midwives in the study by Christoffersen et al.^19^ emphasized that it felt good to attend the funeral of the baby lost at the end of the traumatic birth with the family.

## CONCLUSIONS

Our results showed that midwives who experience traumatic births have similar experiences even in different countries. Midwives who witness the trauma are mostly emotionally affected by this process. They lose their self-confidence and feel a desire to leave their profession. They emphasize the importance of peer support, through which they can share their traumatic experiences.

Psychological education should be primarily provided to midwives who witness the trauma by health professionals. Besides, since all health professionals working in the delivery unit (such as midwives, obstetric nurses, obstetricians) are also at risk of trauma, psychological education should be given to them at regular intervals for their mental health, even if they have not experienced trauma so far. It is seen that birth traumas are generally experienced after a difficult birth. Therefore, in-service training with the most realistic training methods (such as simulation) should be implemented to promote the ability to cope with problems at difficult births, to benefit from the experiences of other midwives, to prevent malpractice, and to reduce occupational burnout. The mother and her family, who have undergone trauma at birth, can experience trauma by committing psychological and physical violence against the midwife. Midwives, therefore, need to be strengthened for the effects of trauma (such as non-pecuniary compensation, depreciation, insurance) both in terms of institutional and midwifery-specific legal policies. In the post-traumatic period, associations supporting all reproductive health, especially midwifery associations, should take a supportive approach to protect the rights of midwives and to protect/ increase their commitment and their sense of belonging to the midwifery profession. To increase midwives’ ability to cope with trauma, to avoid its long-term effects, and to maintain their professional values and autonomy, they should be mentally and socially supported by their colleagues, maternity unit, and institution managers.

## Data Availability

Data sharing is not applicable to this article as no new data were created.
